# Systematic review of statistical methods for safety data in malaria chemoprevention in pregnancy trials

**DOI:** 10.1186/s12936-020-03190-z

**Published:** 2020-03-20

**Authors:** Noel Patson, Mavuto Mukaka, Kennedy N. Otwombe, Lawrence Kazembe, Don P. Mathanga, Victor Mwapasa, Alinune N. Kabaghe, Marinus J. C. Eijkemans, Miriam K. Laufer, Tobias Chirwa

**Affiliations:** 1 School of Public Health, University of the Witwatersrand, Johannesburg, South Africa; 2 University of Malawi, College of Medicine, Blantyre, Malawi; 3 Mahidol Oxford Tropical Medicine Research Unit (MORU), Bangkok, Thailand; 4 Centre for Tropical Medicine, Nuffield Department of Medicine, University of Oxford, Oxford, UK; 5 Perinatal HIV Research Unit, Faculty of Health Sciences, University of the Witwatersrand, Johannesburg, South Africa; 6 Department of Biostatistics, University of Namibia, Windhoek, Namibia; 7 Julius Center for Health Sciences and Primary Care, University Medical Center Utrecht, Utrecht University, Utrecht, The Netherlands; 8 Center for Vaccine Development and Global Health, University of Maryland, School of Medicine, 685 W. Baltimore St., HSF-1 Room 480, Baltimore, MD 21201 USA

**Keywords:** Pregnancy, Malaria, Safety, Prevention, Clinical trials, Statistical methods

## Abstract

**Background:**

Drug safety assessments in clinical trials present unique analytical challenges. Some of these include adjusting for individual follow-up time, repeated measurements of multiple outcomes and missing data among others. Furthermore, pre-specifying appropriate analysis becomes difficult as some safety endpoints are unexpected. Although existing guidelines such as CONSORT encourage thorough reporting of adverse events (AEs) in clinical trials, they provide limited details for safety data analysis. The limited guidelines may influence suboptimal analysis by failing to account for some analysis challenges above. A typical example where such challenges exist are trials of anti-malarial drugs for malaria prevention during pregnancy. Lack of proper standardized evaluation of the safety of antimalarial drugs has limited the ability to draw conclusions about safety. Therefore, a systematic review was conducted to establish the current practice in statistical analysis for preventive antimalarial drug safety in pregnancy.

**Methods:**

The search included five databases (PubMed, Embase, Scopus, Malaria in Pregnancy Library and Cochrane Central Register of Controlled Trials) to identify original English articles reporting Phase III randomized controlled trials (RCTs) on anti-malarial drugs for malaria prevention in pregnancy published from January 2010 to July 2019.

**Results:**

Eighteen trials were included in this review that collected multiple longitudinal safety outcomes including AEs. Statistical analysis and reporting of the safety outcomes in all the trials used descriptive statistics; proportions/counts (n = 18, 100%) and mean/median (n = 2, 11.1%). Results presentation included tabular (n = 16, 88.9%) and text description (n = 2, 11.1%). Univariate inferential methods were reported in most trials (n = 16, 88.9%); including Chi square/Fisher’s exact test (n = 12, 66.7%), *t* test (n = 2, 11.1%) and Mann–Whitney/Wilcoxon test (n = 1, 5.6%). Multivariable methods, including Poisson and negative binomial were reported in few trials (n = 3, 16.7%). Assessment of a potential link between missing efficacy data and safety outcomes was not reported in any of the trials that reported efficacy missing data (n = 7, 38.9%).

**Conclusion:**

The review demonstrated that statistical analysis of safety data in anti-malarial drugs for malarial chemoprevention in pregnancy RCTs is inadequate. The analyses insufficiently account for multiple safety outcomes potential dependence, follow-up time and informative missing data which can compromise anti-malarial drug safety evidence development, based on the available data.

## Background

Drug safety assessment in randomized controlled trials (RCTs) is integral in development of comprehensive drug safety profile. Recently, there is enhanced quality guidance on clinical trial reporting of safety outcomes through adherence to the Consolidated Standards of Reporting Trials (CONSORT) guidelines [1, 2]. However, there is scant literature on standardized ways to statistically analyse the safety outcomes in clinical trials. Although there exist some general regulatory guidelines on safety data analysis, such as International Conference on Harmonization which recommend descriptive statistical methods supplemented by confidence intervals [3, 4], the proposed statistical methods rarely account for the complexity of the collected safety data, e.g., recurrent adverse events (AEs). Effective solutions to statistical analysis of safety data in clinical trials may need to be tailored to specific indications (set of diseases with similar characteristics) since safety data collected are also influenced by the medical condition under study. Absence of standardized guidelines for safety data analysis in specific settings may limit the ability to draw rich conclusions about the safety of the investigational product, based on collected data. Standardized guidelines can simplify integration of safety information from multiple outcomes across RCTs [5] and would ensure optimal use of data in developing the safety profile of the investigational product.

Statistical analysis of safety data in clinical trials is characterized by a challenge of multiple and related endpoints measured over time. The safety endpoints may include clinical and laboratory defined AEs. Laboratory-based AEs are defined based on standard cut-off points for measures such as vital signs (e.g., body temperature), hepato-toxicity measures (e.g., bilirubin level), cardio-toxicity measures (e.g., electrocardiograms), and other tests relevant to the medical indication being studied [5]. The safety endpoints may be correlated within patients and over time such that failure to account for this in an analysis may yield biased estimates and false inference. Furthermore, time to occurrence of the safety endpoint may be very informative in profiling the drug safety. Such data present statistical analysis and interpretation challenges due to the complexity in structure [6]. For instance, in the case of multiple, repeatedly measured, safety outcomes, false positives may arise from multiple statistical testing if appropriate longitudinal or time to event methods and/or multiplicity adjustments are not considered.

In clinical trials, AEs may impact compliance and study participation which may further affect treatment efficacy estimates [7, 8]. Occurrence of (even mild) AEs due to a drug would lead to non-adherence, leading to informative censoring. The dropping of the patients from the study generates missing data that may lead to biased results if poorly accounted for. Therefore, safety data analysis accounting for missing data is useful to facilitate identification and characterization of the safety profile of the drug as early as possible. Other analysis challenges include lack of adequate ascertainment and classification of AEs, and limited generalizability of results [9] since some AEs cannot be pre-specified at study design stage.

There are many populations where drug safety assessment is complex. One of the special settings in safety data assessment is the use of drugs to prevent adverse outcomes in pregnancy, currently referred to as intermittent preventive treatment of malaria in pregnancy (IPTp). For example, the World Health Organization recommends that pregnant women receive routine treatment with anti-malarial drugs to clear any malaria infection that is present and also to prevent infection in the weeks after administration [10]. Recent review indicates that methodological issues in studying anti-malarial drugs in pregnancy have prevented firm conclusions on the safety of new anti-malarial drugs in pregnancy [11]. Previous efforts have attempted to standardize safety assessment methodology for anti-malarial drug trials in pregnancy, including study designs and data collection [12, 13]. However, literature remains limited in describing the standard practice in the statistical analysis of safety data that are collected on anti-malarial drugs during pregnancy trials.

The current review focusses on safety assessment in anti-malarial drugs for chemoprevention in pregnancy trials. Since anti-malarial drug for malaria chemoprevention is given repeatedly to healthy pregnant women, it is critical to improve safety assessment in this vulnerable population. Specifically, appropriate statistical analysis of safety outcomes can improve development of anti-malarial drug safety profile. This can be achieved through sufficient use of the data generated during the RCT which provides a comprehensive drug safety insight. This review, therefore, aims at identifying applied statistical methods and their appropriateness in the analysis of safety data in anti-malarial drugs for malaria prevention during pregnancy clinical trials.

## Methods

The systematic review was conducted according to Preferred Reporting Items for Systematic Review and Meta-Analyses (PRISMA) statement [14] which outlines minimum standards for reporting systematic reviews and meta-analysis (Additional file 1: Table S1). The protocol for this review was registered and published with PROSPERO (CRD42019120916). The study population is pregnant women on any anti-malarial drug for malaria chemoprevention.

### Search strategy

#### Inclusion criteria

Primary original articles published in English from Phase III RCTs were considered for inclusion. The articles were from RCTs assessing the efficacy and safety of malaria chemoprevention in pregnancy. This review focused on Phase III RCTs, because they have the largest sample size among pre-marketing trials and accommodates multidisciplinary support in safety evaluation. Further, the data are systematically collected and have the benefit of being randomized, which aids a fair comparison of treatment groups.

#### Exclusion criteria

Observational studies, case reports, letters to the editor, narrative reviews, systematic reviews and trials in Phase I or Phase II or Phase IV were excluded from this review. This review did not include clinical trials on malaria prevention in pregnancy using intermittent screening and treatment (ISTp) as an alternative to IPTp. ISTp refers to intermittent rapid diagnostic testing (RDT) for malaria in pregnant women followed by treatment of RDT-positive cases with an effective artemisinin-based combination therapy, and IPTp is given to pregnant women regardless of their malaria status. Hence, ISTp and IPTp consider different populations which may confound the practice in safety assessment methods (i.e., ISTp considers symptomatic population and IPTp considers both symptomatic and asymptomatic population). Non-English publications were excluded.

### Selection of studies

Studies published between 1 January 2010 and 31 July, 2019 were searched from five databases (PubMed, Embase, Scopus, Malaria in Pregnancy Libray (MiPL) and Cochrane Central Register of Controlled Trials (CENTRAL). The MiPL is an excellent scholarly source of articles on malaria in pregnancy that enabled the review to capture both indexed and non-indexed articles beyond the searched databases. Additional searches included reference lists of the identified trials and relevant reviews to identify trials potentially missed in the database search. The year 2010 was selected since it is when CONSORT guideline updated and emphasized on appropriate statistical analysis and reporting of clinical trials [15]. Conference proceedings were not included because they usually contain abstracts that do not give detailed analysis of the presented results and they are not rigorously peer-reviewed. The review focussed on published studies only so no experts or abstract publication authors were contacted for unpublished data. The key search items included: *malaria, anti*-*malarial drug, pregnancy, efficacy safety or tolerability.* The detailed search strategy is presented in Additional file 2: Table S2. The search was customized per database. Based on PRISMA procedure, after removing duplicates, two reviewers (NP and ANK) independently screened titles and abstracts initially before arriving at a final list of eligible articles. Based on the eligible studies list, full text articles were retrieved. The references were managed using Endnote X7.1 (Thomson Reuters). If there were disagreements, the two reviewers discussed the paper to reach a consensus and reasons for exclusion were provided for ineligible publications/studies

### Data extraction

The data extraction file created in Microsoft Excel was used to record all key variables from the selected articles. Some of the collected variables such as mode of safety data collection, participant withdrawal due to AE and handling of continuous measures were based on CONSORT guideline. The following key variables were extracted from the papers: main author, publication date, study design, study location, main efficacy outcome, sample size, list of safety parameters collected (including laboratory data), nature of safety data collection (i.e., passive or active), list of statistical methods used for respective safety outcomes, how the results were presented, retention rate at the end of the follow up and how missing safety or efficacy data were handled. The primary hypothesis type (as superiority, non-inferiority or equivalence) was defined based on what was reported in the actual manuscript or inferred by the lead author (NP), based on how the study framed the primary hypothesis. Superiority hypotheses aim to show whether treatment is better than control, non-inferiority hypotheses intend to show that one treatment is not worse than the other and equivalence hypotheses intend to show that a given treatment is similar to another for defined characteristics [16]. The statistical methods were classified as descriptive or inferential and univariate or multivariate depending on the purpose and nature of the statistical methods based on previous similar reviews [17, 18], reviewing statistical methods.

### Data synthesis

The extracted quantitative data were reported as percentages in tables. The commonly reported safety parameters, suitability of the used statistical methods and other findings were also summarized narratively.

## Results

**Fig. 1:**
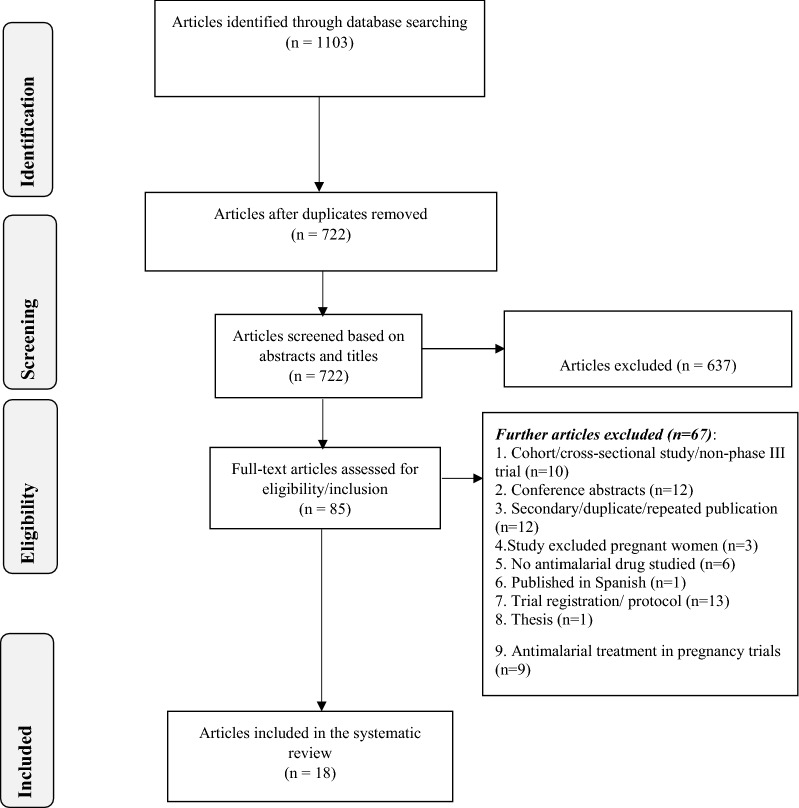
PRISMA flow diagram for study selection process

The search identified 1103 articles. After removing duplicates, 722 unique articles were identified and considered for possible inclusion in the review. The duplicates (i.e. repeated citations) were the same articles but identified in multiple search databases. Figure 1 presents details of the selection process. During screening, a total of 637 articles were excluded based on relevance of their titles and abstracts. The remaining 85 full text articles were assessed for possible inclusion and 18 articles satisfied the inclusion criteria, and were included in this review as shown in Table 1. Reasons for exclusion are shown in Fig. 1.

**Table 1:** Overview of randomized clinical trials included in the systematic review

1st Author, year (reference)	Study site	DSMB	Hypothesis	Masking	Comparator drug(s)	Experimental drug(s)
Luntamo 2010 [39]	Malawi	Yes	Superiority	Single-blinded	Two-dose IPTp-SP	AZ-SP Monthly SP
Valea 2010 [40]	Burkina Faso	No	Superiority	Not stated	Two-dose IPTp	Three IPTp-SP
Diakite 2010 [41]	Mali	Yes	Superiority	Open-label	Two-dose IPTp	Three IPTp-SP
Ndyomugyenyi 2011 [42]	Uganda	Yes	Superiority	Double blinded	IPTp-SPITN + placebo	IPTp-SP + ITN
Wini 2013 [43]	Solomon Islands	Yes	Superiority	Open-label	IPTp-SP	-CQ prophylaxis
Denoeud-Ndam 2014 [44]	Benin	Yes	Noninferiority	Open-label	IPTp-MQ,(IPTp-MQ) and CTX (low CD4)	CTX(low CD4)CTX(high CD4)
González 2014 [45]	Benin, Gabon, Mozambique, and Tanzania	Yes	Superiority	Open-label	IPTp-SP	Two-dose MQ Split-dose MQ
González 2014 [46]	Kenya, Mozambique, and Tanzania	Yes	Superiority		CTX and Placebo	CTX and (IPTp-MQ)
Klement 2014 [47]	Togo	Yes	Noninferiority	Open-label	IPTp-SP	CTX
Manyando 2014 [48]	Zambia	Yes	Noninferiority	Open-label	IPTp-SP	CTX
Desai 2015 [49]	Kenya	No	Superiority	Open-label	IPTp-SP	IPTp-DP IST-DP
Unger 2015 [50]	Papua New Guinea	Yes	Superiority	Single blinded	IPTp-AZCQ	one-dose SP-CQ
Kakuru 2016 [51]	Uganda	No	Superiority	Double blinded	IPTp-SP,	IPTp-DP IPTp–IPTp-DP
Kimani 2016 [52]	Benin, Kenya, Malawi, Tanzania, and Uganda	Yes	Superiority	Open-label	IPTp-SP	IPTp-AZCQ
Natureeba 2017 [53]	Uganda	No	Superiority	Double blinded	TMP-SMX	TMP-SMX + DP
Divala 2018 [54]	Malawi	Yes	Superiority	Open-label	IPTp-SP	IPTp-CQ CQ prophylaxis
Akinyotu 2018 [55]	Nigeria	No	Superiority	Single-blind	IPTp-SP	IPTp-MQ
Kajubi 2019 [56]	Uganda	No	Superiority	Double-blind	IPTpSP	IPTpDP

AL: Artemether-lumefantrine; CTX: Cotrimoxazle; Q: Quinine; AS: Artesunate; AQ: Amodiaquine; MQ: Mefloquine; CQ: Chloroquine; DP: Dihydroartemisinin-piperaquine; TMP-SMX: Trimethoprim-sulfamethoxazole; PQ: Piperaquine

### Characteristics of the trials

The trials included in this review were conducted in Oceania (2 trials, 11.1%) and sub-Saharan Africa (16 trials, 88.9%) regions. The 18 RCTs reviewed recruited 26,281 pregnant women with a median sample size of 374 (interquartile range (IQR): 173, 648) women per treatment arm in a trial. Thirteen trials (72.2%) recruited more than 200 patients per arm. As expected, all trials (18 trials, 100%) computed sample size based on the efficacy outcome(s). The majority of the trials (11 trials, 65.4%) had two treatment arms and the rest had three treatment arms. All 18 trials had an active comparator and IPTp-SP was studied as a standard malaria chemoprevention in the majority of trials (14 trials, 77.8%). Although the review focussed on published trials from 2010 to 2019, the trials were conducted between 2003 and 2017. Based on the primary hypothesis tested, superiority design RCTs were the most common (15 trials, 83.3%) and the other trials had a non-inferiority hypothesis. Over half of the trials (10 trials, 55.6%) were open-label; one trial did not state the blinding status but the other 7 trials (38.9%) were blinded. Majority of the trials (14 trials, 77.9%) reported that they had a Data Safety and Monitoring Board (DSMB).

### Characteristics of the reported safety data

Over half of the trials (10 trials, 55.6%) reported that they collected safety data using a combination of scheduled and non-scheduled visits (Table 2), while a third of the trials (6 trials, 33.3%) did not specify the safety data collection approach used. The median retention rate (based on the defined efficacy outcome reported for respective trials) was 89.4% (IQR: 82.5%, 92.4%) and 10 trials (55.6%) had a retention rate below 90%.

**Table 2:** Characteristics and structure of reported safety data (n = 18)

Characteristic	Number of articles (%)
Data Monitoring and Safety Board	14 (77.8)
Data collection approach	
Scheduled visits	1 (5.6)
Scheduled and non-scheduled visits	10 (55.6)
Non-scheduled visits	1 (5.6)
Not specified	6 (33.3)
Reported AE severity levels	13 (72.2)
Assessed AE vs adherence	3 (16.7)
Discontinue treatment due to AE	18 (100)
Reported continuous outcome dichotomization	18 (100)
Reported laboratory data	18 (100)
Multiple safety endpoints	18 (100)
Longitudinal safety endpoints	18 (100)
Reported recurrent AEs	0 (0.0)
Retention rate (IQR)	89.4% (IQR: 82.5%, 92.4%)

The retention rate is based on primary outcome reported in trial flow diagram

IQR: interquartile range

All the reviewed trials indicated that they had collected multiple longitudinal safety endpoints. As expected, almost all the trials (17 trials, 94.4%) reported obstetric safety outcomes such as foetal loss. Table S3 and S4 in Additional file 2 provide a detailed list of safety outcomes and respective statistical method reported in each reviewed trial. Despite the reported occurrence of multiple AEs, none of trials seemingly reported recurrence of AEs during pregnancy. In total, 12 trials (66.7%) reported adverse events with different severity levels, e.g., mild, moderate and severe. All trials reported occurrence of AEs by treatment arm. Almost all trials (17 trials, 94.4% %) reported laboratory data in their safety assessment of the drug and 16 trials of these (88.9) dichotomized at least a single continuous safety outcome (e.g., haemoglobin) based on standard cut-off points, to define an AE.

### Statistical analysis for safety data analysis

#### Analysis population and missing data

The safety analysis approach (based on treatment allocation and adherence) was specified and reported in 11 trials (61.1%). Per protocol (PP) and intention to treat (ITT) analysis approaches were used in 5 trials (27.7%) and 4 trials (22.2%), respectively. Two trials indicated that they used both PP and ITT to analyse the safety data. Although all the reviewed trials had at least one patient lost to follow-up, only 7 trials (38.9%) reported missing efficacy data and 2 of the 7 trials indicated that the missingness was ignorable after exploring data missingness patterns (Table 3). None of the reviewed trials conducted an advanced sensitivity analysis on the relationship between missing data and drug safety. For example, none of the studies assessed the safety outcomes (e.g., AEs) in relation to missing efficacy outcomes. This review found that most trials (16 trials, 88.9%) had at least one participant who experienced an AE leading to discontinuation from the trial although the studies did not formally investigate/quantify the relationship between the AEs and trial completion.

**Table 3:** Statistical approaches for safety data analysis

Statistical method/approach	Number of articles (%)
Descriptive statistics	18 (100)
Proportion/count	18 (100)
Mean/median	1 (5.6)
Incidence rate	6 (33.3)
Inferential methods	
Univariate methods	
Fisher`s exact test/Chi square	12 (66.7)
t-test	4 (22.2)
Linear regression	1 (5.6)
Mann–Whitney/Wilcoxon	1 (5.6)
Multivariable modelling	
Poisson regression	3 (16.7)
Negative binomial	1 (5.6)
Number of inferential methods	
None	3 (16.7)
One method	10 (55.6)
More than one method	5 (27.8)
Analysis approach	
Reporting missing data	
Missing efficacy data reported	7 (38.9)
Handling missing efficacy data	
Imputation	1 (5.6)
Analysis population definition	
ITT	4 (22.2)
PP	5 (27.8)
ITT + PP	2 (11.1)
Not specified	7 (38.9)
Multiplicity adjustment for treatment	0 (0.0)
Results presentation	
Graphs	0 (0.0)
Tables	16 (88.9)
Point estimate and confidence interval	10 (55.6)
P value	16 (88.9)

Entries in this table are mutually exclusive and may not add up to the total 26 articles since some articles used more than one approach

ITT: intention to treat; PP: per protocol

#### Reported statistical methods

All the trials included reviewed used descriptive statistics as one of the methods to summarize AEs (Table 3). Proportions or counts were the descriptive statistics used in all of the studies to report safety data. Definition safety data was dependent on respective trials as shown in the Additional file 2: Table S3 and S4). Incidence rates were reported in 6 trials (33.3%). Most trials (16 trials, 88.9%) reported univariate inferential statistical methods; these included Chi square or Fisher`s exact test (12 trials, 66.7%), t-test (n = 4, 22.2%). Only 3 trials reported multivariate statistical methods. The multivariable methods were Poisson regression (n = 3, 16.7%), and negative binomial regression (n = 1, 5.6%). Usage of at least two inferential statistical methods to compare safety outcomes was reported in 5 trials (27.8%). Although all studies reported multiple safety outcomes, none reported adjustment for multiplicity during analysis.

The review showed that at least a single optimal statistical methods was reported in 3 trials (16.7%) that considered multivariable modelling. Even though univariable analysis comparing arms in an RCT were appropriately used, further inferential statistical methods reported in the rest of the trials were suboptimal for the type of data being collected. For further details, Additional file 2: Tables S4 provide a detailed list of reported statistical methods with their respective safety outcome(s).

#### Presentation of safety outcomes estimates

In terms of presentation of results, none of the trials presented AEs in a graph. Only 2 trials (11.1%) narratively presented the safety results; the other 16 trials (88.9%) presented the results in tabular format. A total of 14 trials reported p-values after comparing treatments and there were only 10 trials (55.6%) that reported point estimates with their respective confidence intervals.

## Discussion

This review sought to provide a detailed overview of the actual practice of the statistical analysis of safety data in the unique setting of drug trials for the preventions of malaria in pregnancy as reflected published literature. The results demonstrate that there is limited reporting of statistical analyses of safety data, at the end of the trial, in these published reports. The findings are useful to advance the development of standardized guidelines for safety data statistical analysis in analysis in anti-malarial drugs in pregnancy trials and related fields. Such guidelines will not replace but rather complement the CONSORT guidelines that are general (i.e., not providing specific statistical methods in analysing harms in RCTs). Based on the authors’ knowledge of the available literature, this is the first paper to review statistical methods for safety data in anti-malarial drugs in pregnancy.

Descriptive methods were commonly used to summarize safety data. This review found that each clinical trial used at least one descriptive method to summarize safety data. Univariate statistical methods such as Chi square or Fishers exact tests were used in two-thirds of the articles reviewed. Such descriptive statistics and univariate statistical inference ignored useful information such as variability in follow-up time, missing data and correlation (for those trials which had their multiple safety outcomes repeatedly measured). Hence there was inefficient data use during analysis that may lead to a loss of useful information for improved and informative conclusions. Although a third of the reviewed trials attempted to use crude incidence, the analyses failed to adequately account for individual patient follow-up-time and potential confounders.

All trials dichotomized at least a single continuous clinical laboratory safety outcome (i.e., where AE was defined based on standard cut-off points for adult toxicity). Although this aids in providing time-specific drug safety status and easy interpretation, the dichotomized outcome may miss some information on the magnitude of the temporal changes, overtime during the trial. The information loss may lead to reduction in statistical power to detect safety signal if it exists. Valid longitudinal methods (used without restriction on cut-off points) can address the information loss by exploiting potential within-subject correlations for the repeated clinical laboratory measurements [19–21]. Furthermore, the longitudinal methods can provide the basis for developing improved cut-off points tailored to pregnant women in malaria-specific settings. To ensure improved uptake of such methods, future work needs to strive towards making the results from the longitudinal methods feasibly interpretable to the medical practitioners.

Only three studies appropriately used multivariable statistical methods. Adjusting for known prognostic covariates is useful to control for confounding that can be introduced due to imbalance when assessing if treatment is independently associated with safety outcome(s). Of secondary interest, covariate adjustment also preserves type I error [22]. Such adjustment for potential confounders (e.g., age) in safety data analysis are suitable in clinical trials with at least moderate sample size, unlike small sample sizes that lead to unstable estimates. Of specific interest in this review, the Poisson model was more suitable in the context of rare AEs which usually have low event rates [21, 23]. Since Poisson regression assumes a constant rate of occurrence of a rare event, it is not ideal for other multiple transient AEs that were common or recurred and would vary in occurrence overtime [24]. Alternatively, mixed effects models could be considered to characterize the safety events over time since they capture patient-specific effects [25, 26]. Whenever time to AE occurrence information is available, survival analysis models may also be preferred to characterize the time to AE occurrence. For recurrent safety events, that may induce dependence, methods that extend the Cox regression model may be preferred; such models include survival mixed effects models (e.g., frailty models) [4].

Almost half of the reviewed trials did not explicitly define the population on which the safety analysis was based. If per protocol analysis is used to address non-adherence there is potential selection bias since it destroys the balance due to randomization. Although CONSORT recommends ITT, as an alternative for analysis of safety endpoints, non-adherence cannot be explicitly addressed with ITT approach since it ignores dropouts, withdrawal or loss-to-follow up for various reasons including safety concerns; ITT-based inference ignores causal effect of the actual treatment received [27]. Patient withdrawal or dropout due to AEs can induce informative censoring useful in quantifying anti-malarial drug safety. For example, if a patient withdraws due to vomiting after taking an anti-malarial drug, their obstetric efficacy outcomes such as birth weight may appear as missing data. In the context of anti-malarial drug for malaria prevention, even mild AE can lead to drug non-adherence. Since the patient has no disease symptoms, they would judge it less costly for them to discontinue the drug than continue experiencing AEs. Hence, inclusion of information on treatment/trial completion status in relation to anti-malarial safety would enrich development of the safety profile of anti-malarial drug in pregnancy. Although study completion status, anti-malarial drug safety and missing data may be interlinked, missing data received limited attention such that the few trials that considered efficacy missing data did not explicitly explore the potential link. Studying such complex associations requires statistical methods that can appropriately estimate the pathway from the anti-malarial chemoprevention to study completion. Advantageously, methods based on causal inference framework, such as mediation analysis [28–31] could be adapted/extended to assess the influence of the AEs on non-adherence in RCTs.

Despite about three-quarters of the trials reporting p-values after comparing safety outcomes by treatment arms, only about half of the reviewed trials adhered to International Harmonisation Conference Guideline E9 in reporting of confidence intervals in quantifying the safety effect size [3, 4]. Use of confidence interval aids in interpretation of results by providing a measure of precision. Furthermore, graphical displaying of safety data to aid in summarizing of safety data was inadequate. Graphs on safety data have a greater ability to convey insight about patterns, trends, or anomalies that may signal potential safety issues compared to presentation of such data in tabular form only [32]. For example, the graphs could help to visualize frequency and changes in AEs over time by treatment arm. The graphs could further help in assessing assumptions for some statistical methods.

Over three-quarters of the reviewed trials were designed as superiority trials based on efficacy outcomes. Although the statistical approach for safety assessment was mainly on superiority hypotheses (for both the superiority and non-superiority trials), clinical and statistical justification of assessing safety based on superiority hypotheses may be invalid. Superiority hypotheses concentrate on the absence of difference in drug safety effect/risk between or across the treatment arms which may be challenging [16]. For example, when comparing high AE incidences, non-significant difference (when using a superiority hypothesis) would not necessarily translate to a conclusion that a drug is safe and well-tolerated since sometimes all compared treatment arms may have high AE incidence. Perhaps, drug safety evaluation should strive to prove that there is no risk beyond a protocol-defined/hypothesized priori clinical safety margin (i.e., no excessive safety risk). Based on the findings in this work, researchers are encouraged to consider defining safety margins in safety assessment of anti-malarial drugs. Since safety is mostly a secondary outcome, it is not straightforward on how to define a non-inferiority margin and the appropriate analysis population. Currently, it is still unclear and debatable how to implement this, such that further research is needed [5].

Interestingly, over half of the trials were open-label which may influence physician clinical safety assessment on a patient and patient reporting of AEs based on their expectations since they know the treatment assigned. Appropriate reporting of the AEs would be guided by data safety and monitoring boards (DSMB) from early stages of the trial. However, availability of DSMBs in over three-quarters of the trials did not translate to improved reporting and analysis. Therefore, DSMB members should advocate for improved analysis approaches for AEs.

Tabel 4 summarizes recommendations to consider on best practices for safety analyses. This provides a general framework for statistical analysis of safety in malaria chemoprevention in pregnancy trials. As highlighted above, the recommendations assume a context where sample size is moderate or large. For rare events, Bayesian approaches can be considered since they do not depend on asymptotic properties when handling rare events and can incorporate prior/external information [33]. Future research work can further consider adapting/extending recently developed statistical methods for rare disease or small population clinical trials towards analysis of rare safety outcomes in IPTp trials [34–36].

**Table 4:** Recommendations for analyses in malaria chemoprevention in pregnancy trials based on key safety outcomes from the systematic review

Safety outcome type after treatment	Examples	Optimal statistical method(s)
Time to event	Time to infection/infestation occurrence, time to abortion	Survival methods (e.g. Kaplan–Meier plots, log rank test followed by cox regression model)
Longitudinal continuous outcomes	QTc prolongation, haemoglobin, vital signs	Mixed effects linear/nonlinear models
Recurrent events	recurrent hospitalizations, recurrent anaemia, recurrent opportunistic infections	Recurrent event models (e.g. Poisson model, negative binomial, Andersen Gill model)
Safety outcomes with informative missing data,	Treatment switching, Dropout/withdrawal due to AE (i.e. AE-induced non-adherence)	Causal inference methods (e.g. mediation analysis)
Multivariate outcomes	Abortion, miscarriage Haemoglobin, QTc, *alanine aminotransferase level*	Multivariate methods: methods for multiple outcomes (e.g. multivariate regression models based on outcome type), methods for repeated outcomes (e.g. multivariate longitudinal analysis, recurrent events models), advanced graphs
Laboratory measurements with temporal variations	Vital signs, haemoglobin	Advanced graphical methods, multivariate methods
Count	Seizure episodes, dizziness episodes	Incidence rate followed by Models for count data (Poisson regression, negative binomial regression)
Outcomes with zero inflated counts	Seizure episodes, dizziness episodes, nausea episodes	Models for counts (Poisson zero inflated models, negative binomial zero-inflated models)

This review agrees with other similar publications focusing on drug safety assessment in clinical trials that have noted the need for further improvement in the statistical analysis of the safety data [9, 37]. This review concurs with a recent review that has noted that inappropriate handling of multiple test is prevalent, although their review focussed on four high impact journals, AE in general and a short time of review period [38]. Issues raised in this review include time-dependence of AEs, informative censoring due to discontinuation of treatment because of AEs, safety graphs, and repeated occurrence of AEs and multivariate longitudinal structure of laboratory data that yields complex correlation. This is an ongoing work whereby further analysis will be explored to address the identified statistical issues above.

The application of the systematic review protocol in describing the current practice is highly reliable and objective since it exhaustively identified the published anti-malarial drug clinical trials in pregnancy for studied period. However, this review covered only the last decade of publications and may have missed studies published in other languages or that did not appear in during the literature search. Because the trials reported in the publications spanned for a decade, it was difficult to assess temporal trends. This review represents the most comprehensive review of safety data analysis practice for this important indication.

## Conclusion

Although useful safety data are collected in malaria chemoprevention in pregnancy clinical trials, the analysis remains sub-optimal and this hinders definitive conclusions about drug safety in this setting. Descriptive statistical methods and dichotomization of continuous outcomes are predominant which may lead to loss of useful information. The definition of analysis population and informative presentation of results are not standardized. Overall, the results suggest that choice of a statistical method(s) to use should be jointly driven by the scientific question of interest, epidemiological/clinical plausibility of the method and structure of the raw data. Further work in addressing the highlighted gaps can enhance drug safety decisions and conclusions.

## Supplementary information

**Additional file 1.** PRISMA 2009 checklist for the systematic review.

**Additional file 2.** Overview of search strategy, safety outcomes and statistical analysis approaches in the reviewed trials.

## Data Availability

Not applicable.

## Abbreviations

CONSORT: Consolidated Standards of Reporting Trials
AE: Adverse event
RCT: Randomized clinical trial
ECG: Electro-cardiograph
ICH: International Conference on Harmonization
IPTp-SP: Intermittent preventive treatment of malaria in pregnancy using sulfadoxine-pyrimethamine
WHO: World Health Organization
PRISMA: Preferred Reporting Items for Systematic Review and Meta-Analyses
PROSPERO: Prospective Register of Systematic Reviews
CTX: Cotrimoxazle
MiPL: Malaria in Pregnancy Library
CENTRAL: Cochrane Central Register of Controlled Trials
DSMB: Data Safety and Monitoring Board
PP: Per protocol
ITT: Intention to treat
ISTp: Intermittent screening and treatment in pregnancy

## References

[1] Tamminga C, Kavanaugh M, Fedders C, Maiolatesi S, Abraham N, Bonhoeffer J (2013). A systematic review of safety data reporting in clinical trials of vaccines against malaria, tuberculosis, and human immunodeficiency virus. Vaccine..

[2] Moher D, Hopewell S, Schulz KF, Montori V, Gøtzsche PC, Devereaux PJ (2010). CONSORT 2010 Explanation and Elaboration: updated guidelines for reporting parallel group randomised trials. BMJ.

[3] Lewis JA (1999). Statistical principles for clinical trials (ICH E9): an introductory note on an international guideline. Stat Med.

[4] ICH Harmonised Tripartite Guideline (1999). Statistical principles for clinical trials. International Conference on Harmonisation E9 Expert Working Group. Stat Med.

[5] Zink RC, Marchenko O, Sanchez-Kam M, Ma H, Jiang Q (2018). Sources of safety data and statistical strategies for design and analysis: clinical trials. Ther Innov Regul Sci..

[6] Munsaka MS, Peace KE, Chen D-G, Menon S (2018). A question-based approach to the analysis of safety data. Biopharmaceutical Applied Statistics Symposium. Biostatistical Analysis of Clinical Trials.

[7] Leporini C, De Sarro G, Russo E (2014). Adherence to therapy and adverse drug reactions: is there a link?. Expert Opin Drug Saf..

[8] Campbell RT, Willox GP, Jhund PS, Hawkins NM, Huang F, Petrie MC (2016). Reporting of lost to follow-up and treatment discontinuation in pharmacotherapy and device trials in chronic heart failure. Circ Heart Fail..

[9] Singh S, Loke YK (2012). Drug safety assessment in clinical trials: methodological challenges and opportunities. Trials..

[10] WHO. Updated WHO policy recommendation: intermittent preventive treatment of malaria in pregnancy using sulfadoxine-pyrimethamine (IPTp-SP). Geneva, World Health Organization, 2012. https://www.who.int/malaria/publications/atoz/who_iptp_sp_policy_recommendation/en/. Accessed 3 Mar 2020.

[11] D’Alessandro U, Hill J, Tarning J, Pell C, Webster J, Gutman J (2018). Treatment of uncomplicated and severe malaria during pregnancy. Lancet Infect Dis..

[12] Saito M, Gilder ME, Nosten F, Guerin PJ, McGready R (2017). Methodology of assessment and reporting of safety in anti-malarial treatment efficacy studies of uncomplicated falciparum malaria in pregnancy: a systematic literature review. Malar J..

[13] Allen EN, Chandler CIR, Mandimika N, Pace C, Mehta U, Barnes KI (2013). Evaluating harm associated with anti-malarial drugs: a survey of methods used by clinical researchers to elicit, assess and record participant-reported adverse events and related data. Malar J..

[14] Liberati A, Altman DG, Tetzlaff J, Mulrow C, Gotzsche PC, Ioannidis JP (2009). The PRISMA statement for reporting systematic reviews and meta-analyses of studies that evaluate health care interventions: explanation and elaboration. Ann Intern Med.

[15] Schulz KF, Altman DG, Moher D (2010). CONSORT 2010 Statement: updated guidelines for reporting parallel group randomised trials. BMJ.

[16] Lesaffre E (2008). Superiority, equivalence, and non-inferiority trials. Bull NYU Hosp Jt Dis.

[17] Colditz GA, Emerson JD (1985). The statistical content of published medical research: some implications for biomedical education. Med Educ.

[18] Emerson JD, Colditz GA (1983). Use of statistical analysis in the New England Journal of Medicine. N Engl J Med.

[19] Verbeke G, Fieuws S, Molenberghs G, Davidian M (2014). The analysis of multivariate longitudinal data: a review. Stat Methods Med Res.

[20] Rosenkranz GK (2009). Modeling laboratory data from clinical trials. Computat Stat Data Anal..

[21] Gould AL (2015). Statistical methods for evaluating safety in medical product development.

[22] Kahan BC, Jairath V, Doré CJ, Morris TP (2014). The risks and rewards of covariate adjustment in randomized trials: an assessment of 12 outcomes from 8 studies. Trials..

[23] Gam CMB, Tanniou J, Keiding N, Løkkegaard EL (2013). A model for the distribution of daily number of births in obstetric clinics based on a descriptive retrospective study. BMJ Open.

[24] Lawless JF, Nadeau C (1995). Some simple robust methods for the analysis of recurrent events. Technometrics.

[25] Rosenkranz GK (2010). An approach to integrated safety analyses from clinical studies. Drug Inform J..

[26] Kim H, Shults J, Patterson S, Goldberg-Alberts R. (2008) Analysis of adverse events in drug safety: a multivariate approach using stratified quasi-least squares. http://biostats.bepress.com/upennbiostat/papers/art29. Accessed 3 Mar 2020.

[27] Dodd S, White IR, Williamson P (2017). A framework for the design, conduct and interpretation of randomised controlled trials in the presence of treatment changes. Trials..

[28] Robins JM (1994). Correcting for non-compliance in randomized trials using structural nested mean models. Commun Stat Theory Methods..

[29] Frangakis C, Rubin D (1999). Addressing complications of intention-to-treat analysis in the combined presence of all-or-none treatment-noncompliance and subsequent missing outcomes. Biometrika.

[30] Nich C, Carroll KM (2002). Intention-to-treat meets missing data: implications of alternate strategies for analyzing clinical trials data. Drug Alcohol Depend.

[31] Ye C, Beyene J, Browne G, Thabane L (2014). Estimating treatment effects in randomised controlled trials with non-compliance: a simulation study. BMJ Open.

[32] Amit O, Heiberger RM, Lane PW (2008). Graphical approaches to the analysis of safety data from clinical trials. Pharm Stat..

[33] Price KL, Amy Xia H, Lakshminarayanan M, Madigan D, Manner D, Scott J (2014). Bayesian methods for design and analysis of safety trials. Pharm Stat..

[34] Friede T, Posch M, Zohar S, Alberti C, Benda N, Comets E (2018). Recent advances in methodology for clinical trials in small populations: the InSPiRe project. Orphanet J Rare Dis..

[35] Hilgers R-D, Bogdan M, Burman C-F, Dette H, Karlsson M, König F (2018). Lessons learned from IDeAl—33 recommendations from the IDeAl-net about design and analysis of small population clinical trials. Orphanet J Rare Dis..

[36] Mitroiu M, Rengerink KO, Pontes C, Sancho A, Vives R, Pesiou S (2018). Applicability and added value of novel methods to improve drug development in rare diseases. Orphanet J Rare Dis..

[37] Lineberry N, Berlin JA, Mansi B, Glasser S, Berkwits M, Klem C (2016). Recommendations to improve adverse event reporting in clinical trial publications: a joint pharmaceutical industry/journal editor perspective. BMJ.

[38] Phillips R, Hazell L, Sauzet O, Cornelius V (2019). Analysis and reporting of adverse events in randomised controlled trials: a review. BMJ Open.

[39] Luntamo M, Kulmala T, Mbewe B, Cheung YB, Maleta K, Ashorn P (2010). Effect of repeated treatment of pregnant women with sulfadoxine-pyrimethamine and azithromycin on preterm delivery in Malawi: a randomized controlled trial. Am J Trop Med Hyg.

[40] Valea I, Tinto H, Drabo MK, Huybregts L, Henry MC, Roberfroid D (2010). Intermittent preventive treatment of malaria with sulphadoxine-pyrimethamine during pregnancy in Burkina Faso: effect of adding a third dose to the standard two-dose regimen on low birth weight, anaemia and pregnancy outcomes. Malar J..

[41] Diakite OS, Maiga OM, Kayentao K, Traoré BT, Djimde A, Traoré B (2011). Superiority of 3 over 2 doses of intermittent preventive treatment with sulfadoxine-pyrimethamine for the prevention of malaria during pregnancy in mali: a randomized controlled trial. Clin Infect Dis.

[42] Ndyomugyenyi R, Clarke SE, Hutchison CL, Hansen KS, Magnussen P (2011). Efficacy of malaria prevention during pregnancy in an area of low and unstable transmission: an individually-randomised placebo-controlled trial using intermittent preventive treatment and insecticide-treated nets in the Kabale Highlands, southwestern Uganda. Trans R Soc Trop Med Hyg.

[43] Wini L, Appleyeard B, Bobogare A, Pikacha J, Seke J, Tuni M (2013). Intermittent preventive treatment with sulfadoxine-pyrimethamine versus weekly chloroquine prophylaxis for malaria in pregnancy in Honiara, Solomon Islands: a randomised trial. Malar World J..

[44] Denoeud-Ndam L, Zannou DM, Fourcade C, Taron-Brocard C, Porcher R, Atadokpede F (2014). Cotrimoxazole prophylaxis versus mefloquine intermittent preventive treatment to prevent malaria in HIV-infected pregnant women: two randomized controlled trials. J Acquir Immune Defic Syndr.

[45] González R, Mombo-Ngoma G, Ouédraogo S, Kakolwa MA, Abdulla S, Accrombessi M (2014). Intermittent preventive treatment of malaria in pregnancy with mefloquine in HIV-negative women: a multicentre randomized controlled trial. PLoS Med..

[46] Gonzalez R, Desai M, Macete E, Ouma P, Kakolwa MA, Abdulla S (2014). Intermittent preventive treatment of malaria in pregnancy with mefloquine in HIV-infected women receiving cotrimoxazole prophylaxis: a multicenter randomized placebo-controlled trial. PLoS Med..

[47] Klement E, Pitché P, Kendjo E, Singo A, D’Almeida S, Akouete F (2014). Effectiveness of co-trimoxazole to prevent *Plasmodium falciparum* malaria in HIV-positive pregnant women in sub-saharan Africa: an open-label, randomized controlled trial. Clin Infect Dis.

[48] Manyando C, Njunju EM, Mwakazanga D, Chongwe G, Mkandawire R, Champo D (2014). Safety of daily Co-trimoxazole in pregnancy in an area of changing malaria epidemiology: a phase 3b randomized controlled clinical trial. PLoS ONE.

[49] Desai M, Gutman J, L’Lanziva A, Otieno K, Juma E, Kariuki S (2015). Intermittent screening and treatment or intermittent preventive treatment with dihydroartemisinin–piperaquine versus intermittent preventive treatment with sulfadoxine-pyrimethamine for the control of malaria during pregnancy in western Kenya: an open-label, three-group, randomised controlled superiority trial. Lancet.

[50] Unger HW, Ome-Kaius M, Wangnapi RA, Umbers AJ, Hanieh S, Suen CS (2015). Sulphadoxine-pyrimethamine plus azithromycin for the prevention of low birthweight in Papua New Guinea: a randomised controlled trial. BMC Med..

[51] Kakuru A, Jagannathan P, Muhindo MK, Natureeba P, Awori P, Nakalembe M (2016). dihydroartemisinin–piperaquine for the prevention of malaria in pregnancy. N Engl J Med.

[52] Kimani J, Phiri K, Kamiza S, Duparc S, Ayoub A, Rojo R (2016). Efficacy and safety of azithromycin-chloroquine versus sulfadoxine-pyrimethamine for intermittent preventive treatment of *Plasmodium falciparum* malaria infection in pregnant women in Africa: an open-label, randomized trial. PLoS ONE.

[53] Natureeba P, Kakuru A, Muhindo M, Ochieng T, Ategeka J, Koss CA (2017). Intermittent preventive treatment with dihydroartemisinin–piperaquine for the prevention of malaria among HIV-infected pregnant women. J Infect Dis.

[54] Divala TH, Mungwira RG, Mawindo PM, Nyirenda OM, Kanjala M, Ndaferankhande M (2018). Chloroquine as weekly chemoprophylaxis or intermittent treatment to prevent malaria in pregnancy in Malawi: a randomised controlled trial. Lancet Infect Dis..

[55] Akinyotu O, Bello F, Abdus-Salam R, Arowojolu A (2018). Comparative study of mefloquine and sulphadoxine-pyrimethamine for malaria prevention among pregnant women with HIV in southwest Nigeria. Int J Gynaecol Obstet.

[56] Kajubi R, Ochieng T, Kakuru A, Jagannathan P, Nakalembe M, Ruel T (2019). Monthly sulfadoxine-pyrimethamine versus dihydroartemisinin–piperaquine for intermittent preventive treatment of malaria in pregnancy: a double-blind, randomised, controlled, superiority trial. Lancet.

