# Supplementary material for: Systematic review of statistical methods for safety data in malaria chemoprevention in pregnancy trials
Source: Malar J. 2020 Mar 20;19:119. doi: 10.1186/s12936-020-03190-z (PMC7085184; doi:10.1186/s12936-020-03190-z)
Supplement: Supplementary file 2 — Additional file 2. Overview of search strategy, safety outcomes and statistical analysis approaches in the reviewed trials. [file 12936_2020_3190_MOESM2_ESM.docx]

| **Table S2: Search Strategy based on PICOTS framework** | |
| --- | --- |
| Components | Characteristics |
| Population | Pregnant women,  Any geographical location |
| Intervention | Exposed to antimalarial drug for malaria prevention |
| Control | Exposed to placebo or standard antimalarial drug for prevention |
| Outcomes | Safety outcomes e.g. Adverse Events, Lost to follow-up, drop-outs or poor adherence.  Safety assessment statistical methods |
| Timing | January 2010 to July 2019 |
| Setting | Limited to English language |

**Table S3: Description of reported continuous laboratory safety outcomes collected and statistical analysis**

| Author, Year, (reference) | Continuous safety outcome | Dichotomized continuous outcome | ***Outcome:*** Time points collected | Statistical method (outcome) |
| --- | --- | --- | --- | --- |
| Luntamo. 2010 (32) | haemoglobin | yes | ***Haemoglobin*:** 4 week interval, from 28 weeks of gestation age to delivery | None |
| Valea 2010 (35) | vital signs, haemoglobin | Yes | ***Haemoglobin*:** First ANC visit, before SP administration, whenever a malaria infection was suspected and at delivery  ***Vital signs*:** At every antenatal clinic visit | None |
| Diakite. 2011(36) | Haemoglobin, white blood count, | Yes | ***Haemoglobin*:** At enrolment, non-scheduled visit and delivery | Multiple linear regression (haemoglobin) |
| Ndyomugyenyi 2011 (37) | haemoglobin | yes | **Haemoglobin:** At enrolment, 36 weeks of gestation age, delivery | Student’s t-test/Analysis of variance (haemoglobin). |
| Wini 2013 (38) | Haemoglobin, white blood cell | yes | ***Haemoglobin*:** At enrolment, 36 weeks of gestation age  ***White blood cells*:** Not stated | Student’s t-test/Analysis of variance (Haemoglobin) |
| Denoeud-Ndam 2014 (40) | Haemoglobin, full blood count, cd4 count, | Yes | ***Haemoglobin, full blood count, cd4 count:*** At enrolment (16-28 weeks), one month interval and **delivery** | Wilcoxon test (haemoglobin) |
| González 2014 (41) | haemoglobin | yes | ***Haemoglobin*:** enrolment, unscheduled visit and delivery | None |
| González 2014 (42) | haemoglobin | Yes | ***Haemoglobin*:** At enrolment, monthly antenatal clinic visits and unscheduled visits and delivery | None |
| Klement 2014 (43) | Leukocyte count, haemoglobin | yes | ***Haemoglobin*, leucocyte count:** At enrolment, monthly antenatal clinic visits and delivery | None |
| Manyando 2014 (44) | Haemoglobin, glucose, proteins | yes | ***Haemoglobin*:** At enrolment, once in third trimester  ***Glucose*, proteins:** not specified | None |
| Desai 2015 (45) | Haemoglobin, leucocyte, proteineuria | yes | ***Haemoglobin***: At enrolment, monthly antenatal clinic visits, unscheduled visit and delivery  *Leucocyte* *and* *proteinuria*: Not specified | Linear regression (haemoglobin) |
| Unger 2015 (48) | Haemoglobin | yes | ***Haemoglobin*:** at enrolment, unscheduled visit and delivery | Mean at enrolment (haemoglobin) |
| Kakuru 2016 (49) | white-cell count, neutrophil count, platelet count, haemoglobin, alanine aminotransferase level | yes | ***white-cell count, neutrophil count, platelet count alanine aminotransferase level haemoglobin:*** at enrolment, 8 week interval and delivery | Mean at enrolment (white-cell count, neutrophil count, platelet count, haemoglobin, alanine aminotransferase level) |
| Kimani 2016 (50) | Haemoglobin, vital signs, glycosuria, proteinuria | yes | ***Haemoglobin***: Enrolment, 36 weeks of gestation age  *Vital signs, glycosuria, proteinuria:* unscheduled visits | t-test/Analysis of variance (haemoglobin) |
| Natureeba 2017 (53) | Haemoglobin, full blood count,  alanine aminotransferase, QT interval | yes | ***Haemoglobin, full blood count, alanine aminotransferase***: at enrolment, every 8 weeks, delivery  ***QT interval*:** prior to the first daily dose and 3–4 hours after third daily dose after reaching 28 weeks of gestation age (confined to a subsample of 55 participants) | t-test/analysis of variance (QT interval) |
| Divala 2018 (56) | haemoglobin | Yes | **Haemoglobin: At enrolment, every 4 weeks, delivery, unscheduled visit** | None |
| Akinyotu 2018 | **CD4 count** | Yes | ***CD4 count*: At enrolment** | None |
| Kajubi 2019 | Haemoglobin, QTc, alanine aminotransferase | yes | ***Haemoglobin, alanine aminotransferase*:** At enrolment, every 8 weeks, at delivery  ***QT interval***: prior to the first daily dose of study drugs and 3–4 h after the third daily dose, at 20, 28, and 36 weeks gestation | t-test/analysis of variance (QT interval), mean (haemoglobin concentration at enrolment) |

Table S4: Detailed list of categorical safety outcomes collected

| 1^st^ Author, Year, (reference) | **Statistical method(s) used** (outcome) |
| --- | --- |
| Luntamo. 2010 (32) | **Epidemiological statistic** (neonatal or perinatal mortality rate)  **Fishers/chi-square/contingency table** (abortions, stillbirths, severe anaemia and infant deaths) |
| Valea 2010 (35) | **Descriptive statistics** (Neonatal death, stillbirth, preterm birth, anaemia, abortion)  **Poisson** (Neonatal death, stillbirth, preterm birth, anaemia, abortion) |
| Diakite. 2011(36) | **Descriptive** (Icterus, congenital anomaly, fever, vomiting, Pregnancy loss, perinatal or neonatal death, skin rash, jaundice)  **Poisson** (Pregnancy loss, perinatal or neonatal death, anaemia)  **Fishers exact/chi-square** (Icterus, congenital anomaly, fever, vomiting) |
| Ndyomugyenyi 2011 (37) | **Descriptive** (abortion, stillbirth, neonatal death, perinatal death, maternal death, congenital anomaly, haemoglobin, anaemia)  **Fishers exact/chi-square** (abortion, stillbirth, neonatal death, perinatal death, maternal death, congenital anomaly) |
| Wini 2013 (38) | **Descriptive** (pins and needles, vomiting, Stillbirth, skin rash, neonatal death, anaemia, mouth blisters, shortness of breath weakness, drowsiness, numbness)) |
| Denoeud-Ndam 2014 (40) | **descriptive** (abortions, stillbirths, and congenital anomaly, Vomiting, nausea, dizziness, cutaneous reaction and fatigue, neonatal deaths, neonatal infection, Fatigue, preterm birth, fever, cough, rash, pain, anaemia, leukopenia, seizure, psychosis, thrombocytopenia, Gastro enteritis and other adverse events)  **Fishers exact/chi-square** (Vomiting, nausea, dizziness, cutaneous reaction and fatigue (abortions, stillbirths, and congenital anomaly, neonatal deaths, neonatal infection, Fatigue, preterm birth, fever, cough, rash, pain, anaemia, leukopenia, seizure, psychosis, Thrombocytopenia, Gastro enteritis and other adverse events) |
| González 2014 (41) | **Descriptive** (dizziness, vomiting, headache, nausea, asthenia, maternal and neonatal deaths, miscarriages, congenital malformations, anaemia. Haemoglobin, AE by system organ class)  **Fishers exact/chi-square** (dizziness, vomiting, headache, nausea, asthenia, maternal and neonatal deaths, miscarriages, congenital malformations, anaemia, AE by system organ class) |
| González 2014 (42) | **Descriptive** (miscarriages, stillbirths, premature births, and congenital malformations, headache, dizziness, vomiting, nausea)  **Fishers exact/chi-square** (miscarriages, stillbirths, premature births, and congenital malformations, headache, dizziness, vomiting, nausea) |
| Klement 2014 (43) | **Descriptive** (cutaneous reaction, Anaemia, Opportunistic infections, leukopenia, haemoglobin, leucocyte count, stillbirth, preterm birth)  **Fishers exact/chi-square** (cutaneous reaction, Anaemia, Opportunistic infections, leukopenia, stillbirth, preterm birth) |
| Manyando 2014 (44) | **Descriptive** (Infections and infestations, Nervous system and other disorders, stillbirth, preterm birth, abortion, tendinitis, deaths Septicaemia with empetigo, pre-eclampsia,) |
| Desai 2015 (45) | **Descriptive** (abortion or still birth, neonatal death, perinatal death, infant death; Pregnancy, puerperium, and perinatal disorders, Infections and infestations, Gastrointestinal disorders, Surgical and medical procedures, Blood and lymphatic system disorders, Musculoskeletal and connective tissue disorders, Nervous system disorders, Injury, poisoning, and procedural complications, Respiratory, thoracic, and mediastinal disorders, General disorders and administration site conditions, Immune system disorders, Vascular disorders, Congenital, familial, and genetic disorders, Pregnancy, puerperium, and perinatal disorders Pregnancy, puerperium, and perinatal disorders, Infections and infestations, General disorders and administration site conditions, Respiratory, thoracic, and mediastinal disorders, Gastrointestinal disorders )  **Fisher`s exact/chi-square** (abortion or still birth, neonatal death, perinatal death, infant death)  **Poisson** (Pregnancy, puerperium, and perinatal disorders, Infections and infestations, Gastrointestinal disorders, Surgical and medical procedures,Blood and lymphatic system disorders, Musculoskeletal and connective tissue disorders, Nervous system disorders, Injury, poisoning, and procedural complications, Respiratory, thoracic, and mediastinal disorders, General disorders and administration site conditions, Immune system disorders, Vascular disorders, Congenital, familial, and genetic disorders, Pregnancy, puerperium, and perinatal disorders Pregnancy, puerperium, and perinatal disorders, Infections and infestations, General disorders and administration site conditions, Respiratory, thoracic, and mediastinal disorders, Gastrointestinal disorders) |
| Unger 2015 (48) | **Descriptive** (anaemia, maternal death, abortion, still birth, emergency caesarean section, hypertension and other maternal SAEs, preterm birth, congenital anomaly, jaundice, infection, birth asyphixia, meconium aspiration syndrome, cephalohaematomia, dizziness, weakness, vomiting, nausea, pruritus, abdominal pain, headache, diarrhoea, facial swelling, loss of appetite, feeling hot, dyspepsia, other )  **Fishers exact/Chi-square** (anaemia, maternal death, abortion, still birth, emergency caesarean section, hypertension and other maternal SAEs, preterm birth, congenital anomaly, jaundice, infection, birth asyphixia, meconium aspiration syndrome, cephalohaematomia, dizziness, weakness, vomiting, nausea, pruritus, abdominal pain, headache, diarrhoea, facial swelling, loss of appetite, feeling hot, dyspepsia, other ) |
| Kakuru 2016 (49) | **Descriptive** (vomiting, abdominal pain, cough, headache, chills, diarrhoea, malaise, dysphagia, nausea, infection, anorexia, anaemia, congenital anomaly, stillbirth, thrombocytopenia , vaginal bleeding, adverse birth outcomes, white-cell count, neutrophil count, platelet count, haemoglobin, alanine aminotransferase level )  Epidemiologic statistic; incidence rate ( adverse event of any severity) |
| Kimani 2016 (50) | **Descriptive** (deaths, stillbirths, Infections and infestations, Pregnancy, puerperium, and perinatal conditions, Congenital, familial, and genetic disorders, Respiratory, thoracic, and mediastinal disorders, concomitant treatment, vital signs) |
| Natureeba 2017 (53) | **Descriptive (**QT interval, abortion, stillbirth, congenital anomaly, anaemia, vomiting, abdominal pain, cough, headache, anaemia, malaise, diarrhoea, chills, thrombocytopenia, anorexia, congenital anomaly, stillbirth)  **Fishers exact/chi-square** (abortion, stillbirth, congenital anomaly, anaemia, vomiting, abdominal pain, cough, headache, anaemia, malaise, diarrhoea, chills, thrombocytopenia, anorexia, congenital anomaly, stillbirth, altered mental status, elevated ALT level) |
| Divala 2018 (56) | **Descriptive (**dizziness, vomiting, palpitations, headache, nausea, abdominal pain)  **Fishers exact/ chi-square** ( dizziness, vomiting, palpitations, headache, nausea, abdominal pain) |
| Akinyotu 2018 | **Fishers exact/chi-square** (Apgar scores, preterm delivery, nausea, vomiting, headache, gastric pain, dizziness) |
| Kajubi 2019 | **Fishers exact/chi-square** (Fetal or neonatal death, anaemia, QTc prolongation, vomiting, abdominal pain, cough, headache, pyuria, diarrhoea, malaise, congenital anomaly, Thrombocytopenia, altered mental status, elevated alanine aminotransferase, respiratory distress, haemorrhage )  **Negative binomial regression (**Fetal or neonatal death, anaemia**)** |
